# The *q*-G method: A *q*-version of the Steepest Descent method for global optimization

**DOI:** 10.1186/s40064-015-1434-4

**Published:** 2015-10-28

**Authors:** Aline C. Soterroni, Roberto L. Galski, Marluce C. Scarabello, Fernando M. Ramos

**Affiliations:** Laboratory of Computing and Applied Mathematics, National Institute for Space Research, São José dos Campos, Brazil; Satellite Tracking and Control Center, National Institute for Space Research, São José dos Campos, Brazil

**Keywords:** Jackson’s derivative, *q*-Derivative, *q*-Gradient, *q*-G method, 90C26

## Abstract

In this work, the *q*-Gradient (*q*-G) method, a *q*-version of the Steepest Descent method, is presented. The main idea behind the *q*-G method is the use of the negative of the *q*-gradient vector of the objective function as the search direction. The *q*-gradient vector, or simply the *q*-gradient, is a generalization of the classical gradient vector based on the concept of Jackson’s derivative from the *q*-calculus. Its use provides the algorithm an effective mechanism for escaping from local minima. The *q*-G method reduces to the Steepest Descent method when the parameter *q* tends to 1. The algorithm has three free parameters and it is implemented so that the search process gradually shifts from global exploration in the beginning to local exploitation in the end. We evaluated the *q*-G method on 34 test functions, and compared its performance with 34 optimization algorithms, including derivative-free algorithms and the Steepest Descent method. Our results show that the *q*-G method is competitive and has a great potential for solving multimodal optimization problems.

## Background

The history of *q*-calculus dates back to the beginning of the previous century when, based on the pioneering works of Euler and Heine, the English reverend Frank Hilton Jackson developed *q*-calculus in a systematic way (Chaundy 1962; Ernst  2000; Kac and Cheung 2002). His work gave rise to generalizations of special numbers, series, functions and, more importantly, to the concepts of the *q*-derivative (Jackson 1908), or Jackson’s derivative, and the *q*-integral (Jackson 1910). Recently, based on Jackson’s derivative, a *q*-version of the classical Steepest Descent method, called the *q*-Gradient (*q*-G) method, has been proposed for solving unconstrained continuous global optimization problems (Soterroni et al. 2010, 2012). The main idea behind this new method is the use of the negative of the *q*-gradient of the objective function as the search direction. The *q*-gradient is calculated based on *q*-derivatives, or Jackson’s derivatives, and requires a *dilation* parameter *q* that controls the balance between global and local search.

**Fig. 1:**
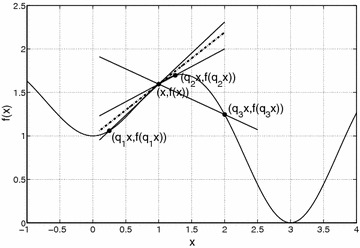
Geometric interpretation of the classical derivative (*dotted line*) and the *q*-derivative for different values of the parameter *q*

The gradient of one-variable function *f*(*x*) is simply the derivative. Geometrically, it is the slope of the tangent line at a given point *x*; see Fig. 1. Similarly, the *q*-gradient of *f* is the *q*-derivative that has also a straightforward geometric interpretation as the slope of the secant line passing through the points [*x*, *f*(*x*)] and [*qx*, *f*(*qx*)]. It is immediately evident that the sign of the *q*-derivative can be either positive or negative, depending on the value of the parameter *q*. For $q_1$ and $q_2$ (Fig. 1), the sign of the *q*-derivative is positive and the *q*-G method will move to the left as the Steepest Descent method would do. However, for $q_3$ the sign of the *q*-derivative is negative which potentially allows the *q*-G method to move to the right direction, towards the global minimum of *f*.

**Fig. 2:**
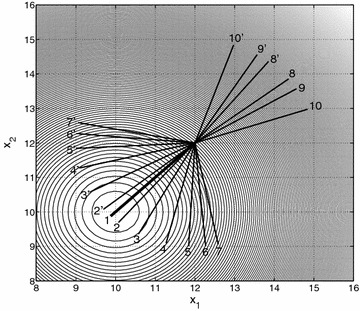
The negative of the gradient vector (*bold line segment* number 1) and the *q*-gradient vector of *f* for different values of the parameter *q* (*line segments* from 2 to 10 and 2′ to 10′). The parameters ($q_1,q_2$) used for each *line segment *are 2: (0.97, 1.03), 2′: (1.03, 0.97), 3: (0.9, 1.1), 3′: (1.1, 0.9), 4: (0.8, 1.2), 4′: (1.2, 0.8), 5: (0.7, 1.3), 5′: (1.3, 0.7), 6: (0.6, 1.4), 6′: (1.4, 0.6), 7: (0.5, 1.5), 7′: (1.5, 0.5), 8: $(-1.2,-0.8)$, 8′: $(-0.8,-1.2)$, 9: $(-1.4,-0.6)$, 9′: $(-0.6,-1.4)$, 10: $(-10,-3)$ and 10′: $(-3,-10)$

Figure 2 shows the contour lines of a quadratic function $f(\mathbf {x})$ with $\mathbf {x} \in \mathbb {R}^2$, shifted to $\mathbf {x}=(10,10)$, and the negative of both classical and *q*-gradient vectors. The negative of the classical gradient vector of *f*, the steepest descent direction, is represented in Fig. 2 by the bold line segment of number 1. All the other line segments illustrate the negative of the *q*-gradient vector obtained for different values of the parameter $\mathbf {q}\in \mathbb {R}^2$, $\mathbf {q}=(q_1,q_2)$. The line segments from 2 to 7 use positive values of $q_1$ and $q_2$ taken symmetrically around 1 [$\mathbf {q}=(q_1,q_2$)], and the line segments from 2’ to 7’ use the same values of $q_1$ and $q_2$, but with inverted positions [$\mathbf {q}=(q_2,q_1)$]. The line segments from 8 to 10 and 8′ to 10′ have a similar description, but use negative values of $q_1$ and $q_2$. Note that as the parameter $\mathbf {q}$ approaches $\mathbf {1}$, the negative of the *q*-gradient tends to the steepest descent direction. As can be seen, depending on the value of the parameter *q*, the *q*-gradient can point to any direction and not only to descent directions.

**Fig. 3:**
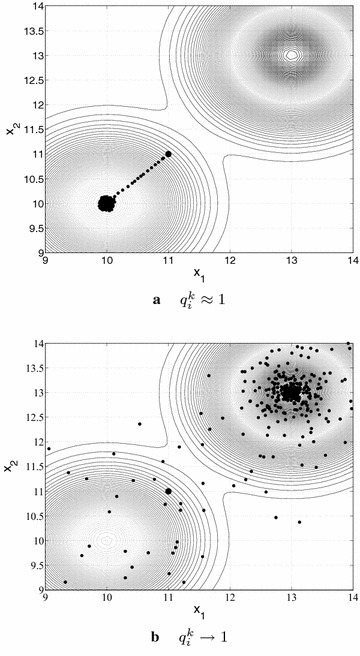
Behavior of the *q*-G method for different values of the parameters $q_i^k$ ($\forall i, \, i=1,2$) along the iterations *k*: **a** $q_i^k \approx 1$ (local search) and **b** $q_i^k \rightarrow 1$ (global search)

Figure 3 illustrates the points sampled by the *q*-G method, with two different strategies to generate the parameter *q*, over a multimodal function $f(\mathbf {x})$, $\mathbf {x} \in \mathbb {R}^2$. The initial point of the search is $\mathbf {x}^0=(11,11)$ and the strategy adopted to define the step length is the same in both cases. Note that the function *f* has a local minimum at $\mathbf {x}=(10,10)$ and a global minimum at $\mathbf {x}=(13,13)$. In Fig. 3a, the parameter $q_i^k$ is fixed and close to 1 along all the iterations *k* ($q_i^k \approx 1$, $\forall i, \quad i=1,2$) and, consequently, the *q*-G method performs a local search converging to the nearest local minimum to the starting point. In Fig. 3b, the parameters $q_i^k$ are different from 1 in the beginning and tend to 1 along the iterative procedure ($q_i^k \rightarrow 1$, $\forall i, \quad i=1,2$), with the *q*-G method performing a global search, with exploration of the search space and exploitation of the global minimum vicinity. As can be seen in Fig. 3, the possibility of having different values of the parameter $q_i^k$, and not only values close to 1, enables the method to move against the local descent direction, escape from the local minimum and sample other regions of the search space. In other words, when the values of $q_i^k$ are sufficiently different from 1, the *q*-gradient vector can point to any region of the search space. This potentially allows the *q*-G method to move towards the global minimum.

These simple examples show that the use of the *q*-gradient, based on Jackson’s derivative, offers a new mechanism for escaping from local minima. The algorithm for the *q*-G method is complemented with strategies to generate the parameter *q* and to compute the step length in a way that the search process gradually shifts from global in the beginning to almost local search in the end. As here proposed, the *q*-G algorithm has only two free parameters to be adjusted: the initial standard deviation ($\sigma ^0$) and the reduction factor ($\beta$). Although a bad choice may lead to some deterioration in its performance, the *q*-G method has shown to be sufficiently robust for still being capable of reaching the global minimum.

We evaluated *q*-G’s performance of against 34 optimization methods and on 34 test problems. First, we considered ten 2-D optimization problems, eight unimodal and two multimodal, as defined in (Luksan and Vlcek 2000), and compared the *q*-G method with 22 derivative-free algorithms described in (Rios and Sahinidis 2013). Second, we evaluated our approach on twelve 10-D and twelve 30-D test problems, ten unimodal and fourteen multimodal. These test problems have been proposed as a benchmark for the CEC′2005 Special Session on Real-Parameter Optimization of the IEEE Congress on Evolutionary Computation 2005 (Suganthan et al. 2005). For this second set of problems, we compared the *q*-G with 11 Evolutionary Algorithms (EAs) participants of the competition and with the steepest descent (SD) method.

The rest of the paper is organized as follows. The "*q*-gradient vector" section introduces the *q*-gradient vector. "The q-G method" section presents an algorithm for the *q*-G method. "Computational experiments" section shows the numerical results, and "Conclusions" section presents our main conclusions.

## The *q*-gradient vector

Let *f*(*x*) be a differentiable one-variable function. The classical derivative of *f* is given by

1 $$\begin{aligned} \frac{df(x)}{dx}=\lim _{\Delta x \rightarrow 0}\frac{f(x+\Delta x)-f(x)}{\Delta x}, \end{aligned}$$

and it is related to infinitesimal summation of the independent variable *x*. The *q*-derivative is related to *dilations* of the independent variable *x* that is multiplied by the parameter *q* as follows (Jackson 1908)

2 $$\begin{aligned} D_qf(x)=\frac{f(qx)-f(x)}{qx-x}, \end{aligned}$$

where $x\ne 0$ and $q\ne 1$. The parameter *q* can be any number different from 1. Therefore, the *q*-derivative can be written as (Koekoev and Koekoev 1993)

3 $$\begin{aligned} D_{q} f(x) = \left\{ \begin{array}{ll} \frac{f(qx) - f(x)}{qx-x}, & \quad x \ne 0 \,\, \text{ and } \,\, q\ne 1 \\ \frac{d f (x)}{dx}, & \quad \text{ otherwise}. \end{array} \right. \end{aligned}$$

Similarly, let $f(\mathbf {x})$ be a differentiable function of *n* real variables, then the first-order partial *q*-derivative of *f* with respect to the variable $x_i$ can be given by (Soterroni et al. 2010)

4 $$\begin{aligned} D_{q_i,x_i} f(\mathbf {x}) = \left\{ \begin{array}{ll} \frac{f(x_1,...,q_ix_i,...,x_n) - f(x_1,...,x_i,...,x_n)}{q_ix_i-x_i}, &{} \quad x_i \ne 0 \,\, \text{ and } \,\, q_i\ne 1 \\ \frac{\partial f(\mathbf {x})}{\partial x_i}, &{} \quad \text{ otherwise}. \end{array} \right. \end{aligned}$$

Thus, the *q*-gradient is the vector of the *n* first-order partial *q*-derivatives of *f* defined as (Soterroni et al. 2010)

5 $$\begin{aligned} \nabla _{\mathbf {q}} f(\mathbf {x})^T = \left[ D_{q_1,x_1} f(\mathbf {x}) \ \ldots \ D_{q_i,x_i} f(\mathbf {x}) \ \ldots \ D_{q_n,x_n} f(\mathbf {x})\right] \end{aligned}$$

where the parameter *q* is a vector $\mathbf {q} = (q_1,\ldots ,q_i,\ldots ,q_n) \in \mathbb {R}^n$. The classical gradient is recovered in the limit of $q_i \rightarrow 1$, for all $i=1,...,n$.

## The *q*-G method

Let a general nonlinear unconstrained optimization problem be defined as

6 $$\begin{aligned} \min f(\mathbf {x}) \end{aligned}$$

where $\mathbf {x} \in \mathbb {R}^n$ is the vector of the independent variables and $f:\mathbb {R}^n \rightarrow \mathbb {R}$ is the objective function. A common optimization strategy is to consider an iterative procedure that, starting from an initial point $\mathbf {x}^0$, generates a sequence $\{\mathbf {x}^k \}$ given by

7 $$\begin{aligned} \mathbf {x}^{k+1}= \mathbf {x}^{k} + \alpha ^{k} \mathbf {d}^{k} \end{aligned}$$

where *k* is the iteration number, $\mathbf {d}^{k}$ is the search direction and $\alpha ^{k}$ is the step length or the distance moved along $\mathbf {d}^{k}$ in the iteration *k*.

Gradient-based optimization methods can be characterized by the different strategies used to move through the search space. The Steepest Descent method, for example, sets $\mathbf {d}^{k} = -\nabla f(\mathbf {x}^{k})$ as the search direction and the step length $\alpha ^{k}$ is usually determined by a line-search technique that minimizes the objective function along the direction $\mathbf {d}^{k}$. In the *q*-G method, the search direction is the negative of the *q*-gradient of the objective function $- \nabla _{q} f(\mathbf {x})$.

Therefore, the optimization procedure defined by (7) becomes

8 $$\begin{aligned} \mathbf {x}^{k+1} = \mathbf {x}^{k} - \alpha ^{k} \nabla _{q} f(\mathbf {x}^{k}). \end{aligned}$$

Key to the performance of the *q*-G method is the correct specification of the parameter *q*. Considering a function of *n* variables $f(\mathbf {x})$, a set of *n* different parameters $q_i \in \mathbb {R}-\{1\}$ ($i=1,\ldots ,n$) are needed to compute the *q*-gradient vector of *f*. The strategy adopted here is to draw the values of $q_ix_i$, $\forall i, \ i=1,\ldots ,n$, from a Gaussian probability density function (pdf), with a standard deviation that decreases as the iterative search proceeds and the mean equal to the current point of the search $x_i$.

**Fig. 4:**
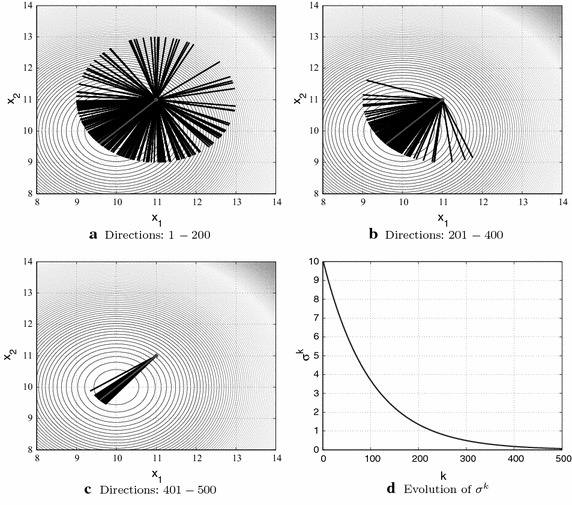
**a**–**c** Possible 500 search directions of the *q*-G method for a 2-D quadratic function at $\mathbf {x}^0=(11,11)$ obtained from parameters *q* generated by a Gaussian distribution with mean $\mu ^k=\mathbf {x}^0$ and standard deviation $\sigma ^k$ that decreases along the iterative procedure from $\sigma ^0=10$ and reduction factor $\beta =0.99$; **d** evolution of the standard deviation $\sigma ^k$ by the expression $\sigma _{k+1}=\beta \cdot \sigma ^k$ used to generate the 500 line segments represented in **a**–**c**. The line segment in *gray* illustrates the steepest descent direction of the function

Starting from $\sigma ^0$, the standard deviation of the pdf is decreased by the following “cooling” schedule, $\sigma ^{k+1} = \beta \cdot \sigma ^{k}$, where $0<\beta <1$ is the reduction factor (see Fig. 4d). As $\sigma ^{k}$ approaches zero, the values of $q_i^k$ tend to 1, the algorithm reduces to the Steepest Descent method, and the search process becomes essentially local. In this sense, the role of the standard deviation here is reminiscent of the one played by the temperature in a Simulated Annealing (SA) algorithm, that is, to make the algorithm sweeps from a global random sampling in the beginning to a local deterministic search in the end. As in a SA algorithm, the performance of the minimization algorithm depends crucially on the choice of parameters $\sigma ^0$ and $\beta$. A too rapid decrease of $\sigma ^{k}$, for example, may cause the algorithm to be trapped in a local minimum.

Figure 4a–c show 500 line segments that represent the possible search directions of the *q*-G method for a 2-D quadratic function at $\mathbf {x}^0=(11,11)$. The parameters *q* were generated by a Gaussian distribution with standard deviation $\sigma ^k$ that decreases along the iterative procedure as $\sigma ^{k+1}=\beta \cdot \sigma ^k$, with a initial standard deviation $\sigma ^0$ and the reduction factor $\beta$. In the beginning of the iterative procedure (Fig. 4a), the search directions can point to anywhere but, as the $\sigma ^k$ tends to 0, the parameter $q_i^k$ tends to 1 and, consequently, the search directions tend to the steepest descent direction (Fig. 4c).

The algorithm of the *q*-G method is completed with a strategy for calculating the step length $\alpha ^k$ based on standard parabolic interpolation. Given the current point of the search $\mathbf {x}^k$ and the parameter $\mathbf {q}^k$, we calculate $\gamma = \Vert \mathbf {q}^k \mathbf {x}^k - \mathbf {x}^k\Vert$ to define the triplet of points $(\mathbf {a},\mathbf {b},\mathbf {c})=(\mathbf {x}^k - \gamma \mathbf {d}^k,\mathbf {x}^k, \mathbf {x}^k + \gamma \mathbf {d}^k )$, where $\mathbf {d}^k$ is the search direction in the iteration *k*. The step length corresponds to the distance between the current value and the minimum value of the parabola passing through $\mathbf {a}$, $\mathbf {b}$ and $\mathbf {c}$. Other strategies for computing the step length are, naturally, possible. Nevertheless, the use of a line-search technique is not recommended since the search directions of the *q*-G method are not always descent directions. Descent directions are required for the success of this kind of one-dimensional minimization (Nocedal and Wright 2006).

Summarizing, the algorithm of the *q*-G method for unconstrained continuous global optimization problems is described as follows.

Algorithm for the*q*-G method

Given $\mathbf {x}^0$ (initial point), $\sigma ^0>0$ and $\beta \in (0,1)$:

1. Set $$k= 0$$
2. Set $$\mathbf {x}_{best} = \mathbf {x}^k$$
3. While the stopping criteria are not reached, do:
  - Generate $\mathbf {q}^k\mathbf {x}^k$ by a Gaussian distribution with $\sigma ^k$ and $\mu ^k=\mathbf {x}^k$
  - Calculate the *q*-gradient vector $\nabla _q f(\mathbf {x}^k)$
  - Set $$\mathbf {d}^{k} = - \nabla _q f(\mathbf {x}^k) / \Vert \nabla _q f(\mathbf {x}^k) \Vert$$
  - Calculate $$\gamma = \Vert \mathbf {q}^k \mathbf {x}^k - \mathbf {x}^k\Vert$$
  - Calculate the triplet $(\mathbf {a},\mathbf {b},\mathbf {c})=(\mathbf {x}^k - \gamma \mathbf {d}^k,\mathbf {x}^k, \mathbf {x}^k + \gamma \mathbf {d}^k )$
  - Compute $\alpha ^k$ by parabolic interpotation, and $\mathbf {x}^{k+1}= \mathbf {x}^{k} + \alpha ^{k} \mathbf {d}^{k}$
  - If $$f(\mathbf {x}^{k+1}) < f(\mathbf {x}_{best})$$ set $$\mathbf {x}_{best} = \mathbf {x}^{k+1}$$
  - Set $$\sigma ^{k+1} = \beta \cdot \sigma ^{k}$$
  - Set $$k = k + 1$$
4. Return $$\mathbf {x}_{best}$$ and $$f(\mathbf {x}_{best})$$.

The *q*-G method stops when the appropriate stopping criterion is attained. In real-world applications (i.e., in problems for which the global minimum is not known), it can be the maximum number of function evaluations, or the value of the local gradient $||\nabla f(\mathbf {x}^k)|| < \epsilon$ ($\epsilon >0$), since the *q*-G method converges to the Steepest Descent method. The algorithm returns the $\mathbf {x}_{best}$ as the minimum value of the objective function *f* obtained during the iterative procedure, i.e., $f(\mathbf {x}_{best}) \le f(\mathbf {x}^k)$, $\forall k$.

## Computational experiments

The *q*-G method was tested on two set of problems followed by a systematic comparison with derivative-free algorithms. First, we applied our approach on ten 2-D test problems defined in (Luksan and Vlcek 2000), eight unimodal and two multimodal, and compared it with 22 derivative-free algorithms described in (Rios and Sahinidis 2013). Second, the *q*-G method was evaluated on twelve 10-D and twelve 30-D test problems, ten unimodal and fourteen multimodal, and it was compared with 11 Evolutionary Algorithms (EAs) participants of the CEC′2005 Special Section on Real-Parameter Optimization of the 2005 IEEE Congress on Evolutionary Computation, and the Steepest Descent method. The *q*-G method and the Steepest Descent method were performed on a iMac 2.7GHz with Intel Core i5 processor and 8GB RAM running Intel Fortran Composer XE for Mac OS* X.

The *q*-G method has two free parameters, the initial standard deviation $\sigma ^0$ and the reduction factor $\beta$. The optimal setting for these parameters is typically problem dependent. The value of $\sigma ^0$ should not be too small so that the algorithm does not behave like its classical version too early, and not too large so that the iterates do not escape the search space frequently. Extensive numerical tests have shown that $\sigma ^0 = \kappa L$, where $\kappa = \sqrt{D/2}$, *D* is the dimension of the problem and *L* is the largest distance within the search space given by $L=\sqrt{\sum _{i=1}^{n} (\mathbf {x}_{{max}_{i}} - \mathbf {x}_{{min}_{i}})}$, provides a simple heuristic for setting this parameter. For example, we set $\sigma ^0 =L$ for all the 2-D problems. This strategy may not provide the best parameter setting for every test function, but is good enough for a wide range of problems.

The value of $\beta$, which controls the speed with which the algorithm shifts from global to local search, also depends on *D*. As a rule of thumb, $\log (1-\beta ) \sim -\kappa$ gives good estimates for $\beta$. For example, for *D* ranging from 1 to 8, $0.9 \le \beta \le 0.99$ is a good choice; for $8 \le D \le 18$, we choose $0.99 \le \beta \le 0.999$; for $18 \le D \le 32$, $0.999 \le \beta \le 0.9999$; and so on. Naturally, the choice of $\beta$ should be balanced with the maximum number of function evaluations of one is willing or limited to perform.

### First set of problems

The first ten 2-D test problems, eight unimodal and two multimodal, are described in Table 1. The evaluation of the objective function at the global optimum $f(\mathbf {x}^{*})$ (last column of Table 1) is used to define a successful run. A run is considered successful if the objective function value at $\mathbf {x}$ is within $1\%$ or 0.01 of the global optimum (Rios and Sahinidis 2013). For each problem, 10 independent runs were performed from random starting points obtained from a uniform distribution within the search space, as defined in (Rios and Sahinidis 2013). The same starting points were used for all methods, including our approach. The stopping criterion used by all algorithms is $N_{max}=2500$ evaluations of the objective function per run.

**Table 1:** A subset of problems prosed at (Luksan and Vlcek 2000) for dimension 2-D

Test problems			$[\mathbf {x}_{min},\mathbf {x}_{max}]^D$	$f(\mathbf {x}^{*})$
Problem 3.1	Rosenbrock	Unimodal	$[-10,000,10,000]^2$	0
Problem 3.2	Crescent	Multimodal	$[-5000,10,000]^2$	0
Problem 3.3	CB2	Unimodal	$[-50,50]^2$	1.9522245
Problem 3.4	CB3	Unimodal	$[-50,50]^2$	2
Problem 3.5	DEM	Unimodal	$[-10,000,10,000]^2$	−3
Problem 3.6	QL	Unimodal	$[-10,000,10,000]^2$	7.20
Problem 3.7	LQ	Unimodal	$[-10000,10000]^2$	−1.4142136
Problem 3.8	Mifflin 1	Unimodal	$[-10,000,10,000]^2$	−1
Problem 3.9	Mifflin 2	Unimodal	$[-10000,10000]^2$	−1
Problem 3.10	Wolf	Multimodal	$[-10,000,10,000]^2$	−8

The column $[\mathbf {x}_{min},\mathbf {x}_{max}]^D$ shows the domain used to define *L*, and the column $f(\mathbf {x}^{*})$ has the evaluation of the function on the global optimum $\mathbf {x}^*$

**Fig. 5:**
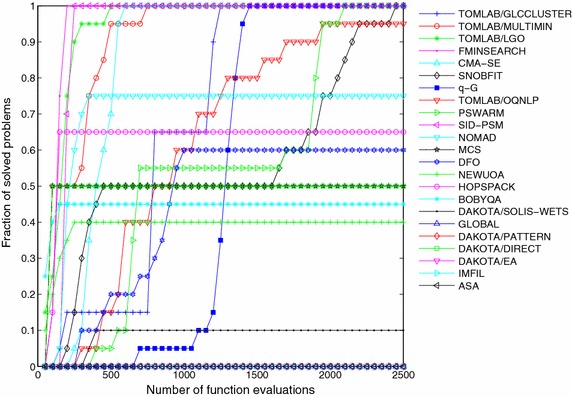
Fraction of multimodal problems solved as function of allowable number of function evaluations

**Fig. 6:**
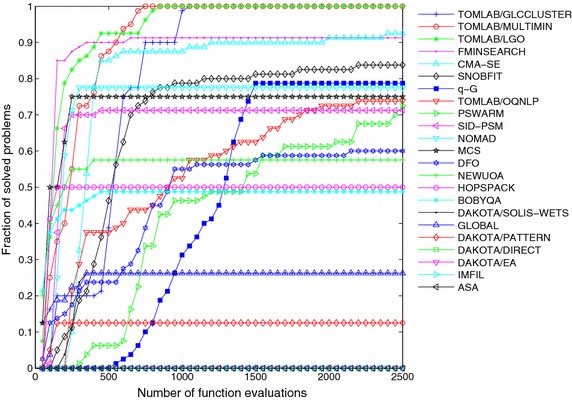
Fraction of unimodal problems solved as function of allowable number of function evaluations

**Fig. 7:**
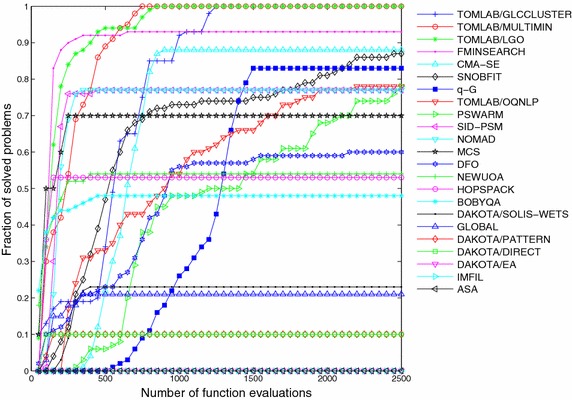
Fraction of all problems solved as function of allowable number of function evaluations

The resolution of all 22 derivative-free methods and the *q*-G method over this set of ten problems, each one solved for 10 independent runs, results in a total number of 100 optimization instances per algorithm. In order to perform a qualitative comparison of the different methods over this set of problems, we calculate for each algorithm the fraction of solved problems given by the ratio between the number of successful runs and the total number of instances. This ratio is computed every 50 evaluations of the objective function. Figures 5, 6 and 7 show the fraction of multimodal, unimodal and all problems, respectively, solved by each method to reach the optimality tolerance along the iterative procedure.

The *q*-G method solved 100 % of the multimodal problems (see Fig. 5), 79 % of the unimodal problems (see Fig. 6) and, in total, our approach solved 83 % of the problems arriving in a 7th position among the 23 methods. The comparison was performed under the same initial conditions and stopping criterion, and the *q*-G method used the same set of free parameters for all problems, namely $\sigma ^0 = L$ and $\beta = 0.95$.

### Second set of problems

The performance of the *q*-G method was also evaluated on twelve 10-D and twelve 30-D test problems, ten unimodal and fourteen multimodal. Their characteristics are summarized in Table 2. These functions are a subset of the problems proposed at CEC′2005 (Suganthan et al. 2005). The *q*-G method is compared with the top eleven Evolutionary Algorithms (EAs) participants of the competition: BLX-GL50 (Garcia-Marinez and Lozano 2005), BLX-MA (Molina et al. 2005), CoEVO (Posik 2005), DE (Ronkkonen et al. 2005), EDA (Yuan and Gallagher 2005), DMS-L-PSO (Liang and Suganthan 2005), L-SaDE (Qin and Suganthan 2005), G-CMA-ES (Auger and Hansen 2005a), L-CMA-ES (Auger and Hansen 2005b), K-PCX (Sinha et al. 2005) and SPC-PNX (Ballester et al. 2005). In addition, we applied the Steepest Descent (SD) method to the same test problems in order to compare the *q*-G method with its classical version.

**Table 2:** A subset of the test problems proposed at CEC′2005 for dimensions 10-D and 30-D

Test problems			$[\mathbf {x}_{min},\mathbf {x}_{max}]^D$	$f(\mathbf {x}^{*})$
Unimodal				
$f_1$		Shifted sphere function	$[-100,100]^D$	−450
$f_2$		Shifted Schwefel’s problem 1.2	$[-100,100]^D$	−450
$f_3$		Shifted rotated high conditioned elliptic function	$[-100,100]^D$	−450
$f_4$		Shifted Schwefel’s problem 1.2 with noise in fitness	$[-100,100]^D$	−450
$f_5$		Schwefel’s problem 2.6 with global optimum on bounds	$[-100,100]^D$	−310
Multimodal				
$f_6$		Shifted Rosenbrock’s function	$[-100,100]^D$	390
$f_7$		Shifted rotated Griewank’s function without bounds	–	−180
$f_9$		Shifted Rastrigin’s function	$[-5,5]^D$	−330
$f_{10}$		Shifted rotated Rastrigin’s function	$[-5,5]^D$	−330
$f_{11}$		Shifted rotated Weierstrass function	$[-0.5,0.5]^D$	90
$f_{12}$		Schwefel’s problem 2.13	$[-\pi ,\pi ]^D$	−460
$f_{15}$		Hybrid composition function	$[-5,5]^D$	120

The column “$[\mathbf {x}_{min},\mathbf {x}_{max}]^D$” shows the domain used to generate the initial points of the search, where *D* is the dimension. The column $f(\mathbf {x}^*)$ shows the evaluation of the function on the global optimum $\mathbf {x}^*$. A complete description of these problems can be found in (Suganthan et al. 2005)

The test problems proposed at CEC′2005 are based on classical benchmark functions such as Sphere, Rosenbrock’s, Rastrigin’s, Ackley’s and Griewank’s function. They contain difficulties such as huge number of local minima, shifted global optimum, rotated domain, noise, global optimum outside the initialization range or within a very narrow basin, and a combination of different function properties (Suganthan et al. 2005). The function $f_{15}$, for example, is a composition of Rastrigin’s, Weierstrass, Griewank’s, Ackley’s and Sphere functions. The selected subset of the CEC′2005 test problems comprises those for which at least one of the thirteen algorithms (eleven EAs, the *q*-G and SD methods) was capable to achieve a fixed accuracy or a target function value defined in (Suganthan et al. 2005).

To ensure a fair comparison, we applied on the *q*-G and SD methods the same evaluation criteria defined in (Suganthan et al. 2005) and used by the EAs. For each function and dimension, the algorithms performed 25 independent runs from different starting points generated with a uniform random distribution within the search space^1^ (see column $[\mathbf {x}_{min},\mathbf {x}_{max}]^D$ of Table 2). The stopping criteria are either the termination error value equal to $10^{-8}$ or less {i.e., $[f(\mathbf {x}_{best})-f(\mathbf {x}^{*})] < 10^{-8}$, where $\mathbf {x}^{*}$ is the global optimum} or the maximum number of function evaluations ($Max\_FEs$) equal to $10{,}000 \times D$. More details of the functions and evaluation criteria can be found in (Suganthan et al. 2005). The resolution of these twelve 10-D and twelve 30-D problems, each one solved for 25 independent runs, results in a total number of 600 optimization instances per algorithm. Here we are also using fixed free parameters $\sigma ^0 = \sqrt{5}L$ with $\beta = 0.995$ for all 10-D problems, and $\sigma ^0 =\sqrt{15}L$ with $\beta = 0.9995$ for all 30-D problems. The SD method uses golden section technique to generate the step length. For the multimodal functions, whenever the SD method terminates before reaching the termination error and the number of function evaluations is less than $Max\_FEs$, it is restarted from a randomly generated point inside the search space.

To evaluate the performance of the methods on this second set of problems, we computed a “success rate” (*SR*) and a “success performance” (*SP*) for each algorithm and function as (Suganthan et al. 2005)

9 $$\begin{aligned} SR=\frac{\text{ number } \text{ of } \text{ successful } \text{ runs }}{\text{ number } \text{ of } \text{ total } \text{ runs }}, \end{aligned}$$

10 $$\begin{aligned} SP=\frac{\text{ mean } \text{ of } \text{ FEs } \text{ for } \text{ successful } \text{ runs }\times \text{ number } \text{ of } \text{ total } \text{ runs }}{\text{ number } \text{ of } \text{ successful } \text{ runs }}. \end{aligned}$$

*SR* represents the fraction of runs that were successful. A successful run for this set of problem is defined as a run in which the algorithm achieves a given accuracy level^2^ with less or equal $Max\_FEs$ (Suganthan et al. 2005). *SP* is the number of function evaluations needed for an algorithm to achieve the minimum, with a given accuracy, in a successful run. Table 3 presents *SR* and *SP* values obtained by the *q*-G and SD methods, for all the twelve 10-D and twelve 30-D test functions.

**Table 3:** Success rate (*SR*) and success performance (*SP*) of the *q*-G and the SD methods for each function and dimension

Functions	10-D				30-D			
	*q*-G		SD		*q*-G		SD	
	*SR*	*SP*	*SR*	*SP*	*SR*	*SP*	*SR*	*SP*
$f_1$	1.00	2.83e+3	1.00	4.68e+1	1.00	1.82e+3	1.00	8.80e+1
$f_2$	0.96	4.15e+4	1.00	3.28e+4	0	–	0.04	7.39e+6
$f_3$	0	–	0	–	0	–	0	–
$f_4$	0.96	3.99e+4	0	–	0	–	0	–
$f_5$	0	–	0	–	0	–	0	–
$f_6$	0	–	0	–	0	–	0	–
$f_7$	1.00	1.22e+4	0	–	1.00	9.28e+4	1.00	2.28e+4
$f_9$	1.00	2.08e+4	0	–	0.88	1.69e+4	0	–
$f_{10}$	1.00	2.69e+4	0	–	0.96	2.57e+4	0	–
$f_{11}$	0	–	0	–	0	–	0	–
$f_{12}$	0	–	0.08	3.55e+5	0	–	0	–
$f_{15}$	0	–	0	–	0	–	0	–

The performance comparison of the *q*-G method with its classical version (SD method) and the top eleven EAs of CEC′2005 was made for two groups: the five unimodal and the eight multimodal test functions. For each group and dimensions, 10-D and 30-D, we calculated the average success rate and the average success performance of each algorithm. The average success rate of an algorithm is the arithmetic mean of the *SP* values in a group of functions. The average success performance is the arithmetic mean of the *SP* values for the functions with at least one successful run. Tables 4 and 5 show the resulting ranking of the algorithms for the unimodal and multimodal functions, respectively, and the dimensions 10-D and 30-D. The algorithms are ranked by the following criteria:

1. Highest value of the average success rate (column “Average *SR*  %”).
2. Number of solved functions in each group (column “SF”). *A function is considered solved by an algorithm if at least one of the runs is a successful run or if the SR is different from 0*.
3. Lowest value of the average success performance (column “Average *SP*”).

**Table 4:** Rank of the algorithms for the unimodal problems and dimensions 10-D and 30-D

10-D				30-D			
Algorithms	Average	SF	Average	Algorithms	Average	SF	Average
	*SR * (%)		*SP*		*SR* (%)		*SP*
G-CMA-ES	100	5	3.85e+03	G-CMA-ES	88	5	3.70e+04
EDA	98	5	1.46e+04	L-CMA-ES	80	4	3.32e+04
DE	96	5	5.68e+04	EDA	80	4	1.81e+05
L-CMA-ES	86	5	4.20e+04	DMS-L-PSO	57	3	1.57e+05
BLX-GL50	80	4	3.22e+04	SPC-PNX	53	3	2.36e+05
CoEVO	80	4	3.53e+04	L-SaDE	50	3	2.36e+05
SPC-PNX	80	4	2.72e+04	K-PCX	40	2	7.53e+03
DMS-L-PSO	76	4	3.75e+04	BLX-GL50	40	2	1.09e+05
L-SaDE	72	4	2.96e+04	SD	21	2	3.70e+06
BLX-MA	59	3	4.10e+04	q-G	20	1	4.51e+04
*q-G*	*59*	*3*	*2.80e+04*	BLX-MA	20	1	3.17e+04
K-PCX	57	3	2.01e+04	DE	20	1	1.39e+05
SD	40	2	1.64e+04	CoEVO	9	2	1.11e+06

**Table 5:** Rank of the algorithms for the multimodal problems and dimensions 10-D and 30-D

10-D				30-D			
Algorithms	Average	SF	Average	Algorithms	Average	SF	Average
	*SR *(%)		*SP*		*SR* (%)		*SP*
G-CMA-ES	69	6	7.53e+04	G-CMA-ES	41	6	1.41e+06
L-SaDE	59	5	6.06e+04	q-G	41	3	4.51e+04
DMS-L-PSO	54	5	5.18e+04	K-PCX	35	5	2.07e+05
K-PCX	47	4	2.99e+04	DMS-L-PSO	30	3	6.33e+05
*q-G*	*43*	*3*	*5.67e+03*	L-CMA-ES	29	2	3.56e+04
DE	39	6	6.91e+05	BLX-GL50	29	2	1.38e+05
BLX-GL50	29	4	9.42e+04	L-SaDE	23	2	1.17e+05
L-CMA-ES	35	3	3.64e+04	SD	14	1	2.28e+04
EDA	21	4	1.68e+05	EDA	14	1	1.31e+05
BLX-MA	13	2	1.87e+05	DE	13	1	2.00e+05
SPC-PNX	1	2	1.45e+05	SPC-PNX	10	2	2.79e+05
SD	1	1	3.55e+05	CoEVO	6	1	5.69e+05
CoEVO	0	0	–	BLX-MA	5	1	6.58e+05

For the unimodal problems, the *q*-G method does not perform very well arriving in eleventh and tenth positions for dimensions 10-D and 30-D, respectively. The average success rates for the unimodal problems are 59 % for 10-D and 20 % for 30-D. Note that the increase of the dimension affected the performance of all algorithms, in terms of either *SR* or the number of solved functions. Overall, the performance of the *q*-G method is not very different from its classical version, the SD method, which arrives in the thirteenth and ninth positions, for dimensions 10-D and 30-D, respectively.

This picture changes for the multimodal problems, where the *q*-G method performed well, arriving in fifth and second positions for 10-D and 30-D, respectively. The average success rates of the *q*-G method are 43 and 41 % for 10-D and 30-D, respectively. As expected, the SD method has a poor performance over the multimodal problems arriving in twelfth and eighth positions for dimensions 10-D and 30-D, respectively. Again, the increase of the dimension affected the performance of all algorithms.

## Conclusions

In this paper we presented the *q*-G method, a generalization of the Steepest Descent method based on the use of the *q*-gradient vector to compute the search direction. This strategy provides the algorithm an effective mechanism for escaping from local minima. As implemented here, the search process performed by the *q*-G method gradually shifts from global search in the beginning to local search in the end. Our computational results have shown that the *q*-G method is competitive and promising. For the multimodal functions in the two set of problems, it performed well compared to the other derivative-free algorithms, some considered to be among the state-of-the-art in the evolutionary computation and numerical optimization communities.

Although our preliminary results show that the method is effective, further research is necessary. Currently, a novel version of the *q*-G method, which is able to guarantee the convergence of the algorithm to the global minimum in a probabilistic sense, is under development. This version is based on the generalized adaptive random search (GARS) framework for deterministic functions (Regis 2010). In addition, gains in the performance of the *q*-G method are expected with the implementation of several improvements, such as inclusion of side, linear and nonlinear restrictions, development of better step selection strategies and others.

## Footnotes

1 For the Shifted rotated Griewank’s function ($f_7$) the domain used to generate the initial points is $[0,600]^D$, where *D* is the dimension of the problem.

2 The accuracy level is defined for each function in (Suganthan et al. 2005).
